# Comparing Activity and Participation between Acquired Brain Injury and Spinal-Cord Injury in Community-Dwelling People with Severe Disability Using WHODAS 2.0

**DOI:** 10.3390/ijerph17093031

**Published:** 2020-04-27

**Authors:** Seo Yeon Yoon, Ja-Ho Leigh, Jieun Lee, Wan Ho Kim

**Affiliations:** 1 Department of Rehabilitation Medicine, Bundang Jesaeng General Hospital, Seongnam, Gyeonggi-do 13590, Korea; 2 Department of Rehabilitation Medicine, Seoul National University Hospital, College of Medicine, Seoul National University, Seoul 03080, Korea; 3 Department of Rehabilitation Medicine, Korea Workers’ Compensation and Welfare Incheon Hospital, Incheon 21417, Korea; 4 Department of Health Convergence, Graduate School of Ewha Womans University, Seoul 03760, Korea; 5 Department of Rehabilitation Medicine, National Rehabilitation Center, Seoul 01022, Korea

**Keywords:** brain injury, spinal-cord injury, disability, World Health Organization Disability Assessment Schedule 2.0 (WHODAS 2.0)

## Abstract

Central-nervous-system (CNS) injuries constitute a significant cause of morbidity (often resulting in long-term disability) and mortality. This cross-sectional study compared the activity and participation of community-dwelling people with severe disability from acquired brain injuries (ABI) (*n* = 322) and spinal-cord injuries (SCI) (*n* = 183) to identify risk factors related to disability. Data were collected through a questionnaire survey of community-dwelling people with severe disability attending 65 healthcare centers. The survey included the World Health Organization Disability Assessment Schedule 2.0 (WHODAS 2.0) and sociodemographic factors. We categorized a registered grade of disability of 1 or 2 as severe disability. WHODAS 2.0 domain and summary scores were compared between the ABI and SCI groups, and risk factors associated with disability were identified through regression analysis. ABI participants had significantly higher disability in cognition and relationships, whereas patients with SCI had higher disability in mobility (*p* < 0.05). Onset duration was negatively correlated with cognition, relationships, participation, and summary scores in ABI participants (*p* < 0.05). Neither group’s socioeconomic factors were associated with WHODA 2.0 scores. Understanding the different patterns of disability between SCI and ABI in community-dwelling people with severe disability helps establish future plans for the management of health resources.

## 1. Introduction

Neurological conditions are among the leading causes of disability and are associated with a substantial burden on patients, their families, and the public health system [1,2]. Of the various neurological conditions, brain injuries such as stroke, traumatic brain injury (TBI), Parkinson’s disease (PD), and spinal-cord injury (SCI) constitute major causes of morbidity and mortality. 

The incidence of stroke per one million persons with income in South Korea was 2650, while that of SCI was 50 [3,4]. Injuries involving the central nervous system (CNS) can cause functional impairment and long-term disability. For instance, many patients have remaining disabilities after a stroke, with life-long consequences for their functional ability and quality of life (QOL), despite relatively successful acute management and advanced multidisciplinary treatments [5,6]. SCI, another common type of CNS injury, is also a severely disabling condition that leads to a range of impairments and decreased QOL [7].

There are numerous instruments for evaluating the severity and outcomes of patients with CNS injuries, including brain injury and SCI [8,9,10,11]. Although disease-specific instruments might be useful in the follow-up of one patient or a group of patients, they do not facilitate the comparison of patients who have different diagnoses that contribute to their disability. The World Health Organization Disability Assessment Schedule 2.0 (WHODAS 2.0), published by the World Health Organization (WHO) in 2010, is a generic assessment tool for health and disability that can be used to produce standardized disability levels [12]. Developed by the International Classification of Functioning, Disability, and Health (ICF), WHODAS 2.0 provides a framework for evaluating six domains of functioning, including social participation and cognition-related daily activities [13]. According to the ICF, “functioning” is an umbrella term encompassing all body functions, activities, and participation, and “disability” serves as an umbrella term for impairments, activity limitations, and participation restrictions [13]. 

Many previous studies focused on the medical problems and treatment of CNS injuries [14,15], while measures of activity and participation for community-dwelling people with chronic CNS injuries remain relatively underdeveloped. Several studies evaluated the activity and participation of people with CNS injuries, and compared the pattern of disability between different disease groups [16,17,18,19,20]. Some previous studies employed WHODAS 2.0 to study various health conditions, including stroke, TBI, SCI, and aging. Two studies compared functioning and disability between TBI and SCI groups [16,17], and another compared the disability level between amyotrophic-lateral-sclerosis and TBI groups [18]. Studies with institutionalized individuals suggested that institution-dwelling is related to poor functioning [16,18]. The mean onset duration in previous studies was approximately seven years [21,22]. Patterns of participation and activity might vary according to the type of disease, severity of disability, onset duration, and socioeconomic factors. 

In Korea, disabilities are classified into 15 groups, and diagnosis is made by the respective medical specialist. Of the 15 disability groups, CNS injuries are divided into two groups: SCI is classified as a physical disability, while disabilities arising from the brain such as stroke, TBI, hypoxic–ischemic brain injury, and PD are classified as brain impairments. The severity of disability is graded from 1 to 6, indicating very severe to very mild disability, on the basis of functional losses and clinical impairment. 

The disability-grading system for SCI is based on the manual muscle test, whereas that for ABI is based on gait function and activities of daily living. Numerous public healthcare plans for people with disabilities have been equally applied to CNS injury patients with different disease groups. For example, a rehabilitation program incorporating neurodevelopment therapy was equally provided on the basis of medical insurance for both ABI and SCI groups. People with severe disability generate a higher burden for society and require substantial health-related resources throughout their lives. Thus, understanding the differences in patterns of disability between the two groups, and applying this understanding to healthcare plans may yield important benefits for health-resource management.

This study sought to evaluate differences in the patterns of disability in people with severe CNS injuries from different causes. The objective of this study was to compare the activity and participation of community-dwelling people with severe disability of different origins, acquired brain injury (ABI) or SCI, and identify risk factors related to disability.

## 2. Materials and Methods

### 2.1. Study Design

This community-based cross-sectional study was conducted in public-healthcare centers (PHCs) in Korea from March to May 2013. Officers who worked in community-based rehabilitation programs in each PHC were trained to administer the 36-item version of WHODAS 2.0 in Korean via face-to-face interviews with home-dwelling people with severe disability. A written questionnaire survey was also administered. The study collected the following sociodemographic information: age, gender, duration of disability, area of residence, type of household, cohabitation with a partner, occupational status, and economic status. The ethics review board of the Korean National Rehabilitation Center approved this study (IRB No. NRC-2012-06-041).

### 2.2. Study Population

We enrolled participants with ABI and SCI aged 20 or older who had lived in the district for more than 2 years. Participants with stroke, TBI, hypoxic–ischemic brain injury, and parkinsonism were enrolled for the ABI group, while participants with myelopathy and traumatic SCI for the SCI group. The detailed International Classification Disease code for each disease is available online (Supplementary Table S1). This study excluded individuals who were admitted to any medical facility within 3 months, newly diagnosed with neurologic disorders that could affect their activity/participation within 3 months, and those who had cerebral palsy or congenital problems. Participants were individuals who registered for community-based rehabilitation services (approximately 5% of the disabled population in the region of each of the 65 nationally evenly distributed PHCs). Participants at each PHC were considered a cluster and randomly contacted to recruit 20 participants per cluster during the 2-month study period. When considering the characteristics of the established cluster, it was necessary to consider the possibility that the target population of this study had a higher level of activity and participation than the general population with ABI and SCI.

To compare the activity and participation of individuals with severe ABI and SCI, this study focused on people with severe disability, designated as Grade 1 or 2 in the Korean National Classification System of People with Disabilities. The study sample included two groups: ABI with severe disability and SCI with severe disability. Participants answered sociodemographic questions and completed the WHODAS 2.0. 

A total of 1340 community-dwelling people with disabilities received the questionnaire, and 1288 (96.1%) responded. Excluding responses with missing and invalid data, 967 (72.1%) responses were eligible for analysis. Among the eligible respondents, 505 matched the category of persons with severe disability: 322 in the ABI group and 183 in the SCI group. Age was divided into two groups according to the aging concept of the United Nations: under 65 and 65 or older [23].

### 2.3. WHODAS 2.0

WHODAS 2.0 was used to measure participant activity and participation. It has high internal consistency (Cronbach’s alpha: 0.86), high test–retest reliability (intraclass correlation coefficient 0.98), and good responsiveness [12]. We followed the manual for WHODAS 2.0 to calculate the summary scores that range from 0 to 100, with 0 meaning no disability and 100 meaning fully disabled. For the ICF classification of disability, WHODAS scores were transformed into ICF disability categories: no problem (0–4%), mild problem (5–24%), moderate problem (25–49%), severe problem (50–95%), and extreme problem (95–100%). Participants with severe or extreme problems were defined as severely disabled on the basis of previous studies [24,25]. 

### 2.4. Statistical Analysis

Baseline categorical variables, expressed as numbers and percentages, were compared between the groups using the chi-squared test, while Student’s t-test was used to compare means for continuous baseline variables, summary, and subscores of WHODAS 2.0. We explored the effects of age and onset duration on WHODAS 2.0 scores by performing linear regression analysis. After comparing the domain and summary scores of WHODAS 2.0 between the two groups, regression analysis was performed to determine the risk factors associated with disability. We estimated the adjusted odds ratio (OR) and 95% confidence interval (CI) for the relationships between socioeconomic factors and severe disability, measured using the ICF classification, by applying a multivariate-logistic-regression model. Statistical significance was considered for *p* < 0.05. All statistical analyses were performed using SAS for Windows, version 9.4 (SAS Institute Inc., Cary, NC, USA).

## 3. Results

Table 1 presents the general characteristics of the participants. Participants under 65 years old made up 59.5% of the ABI group and 70.2% of the SCI group (*p* = 0.017). The onset duration for participants in the SCI group was almost double that for the ABI group (*p* = 0.001). There were also significant differences between the two groups according to area of residence, and marital, occupational, and economic status (*p* < 0.05). The majority of participants in both groups were unemployed. 

Table 2 shows the domain and summary scores of WHODAS 2.0 according to group. The domain scores for cognition and relationships indicated a significantly higher level of disability in the ABI group than that in the SCI group, whereas mobility scores indicated a higher level of disability in the SCI group than that in the ABI group (*p* < 0.05). There were no significant differences in the self-care, life activities, or participation scores, or summary score of WHODAS 2.0 between the two groups (*p >* 0.05).

Regarding age (Table 3), for participants aged 65 years or older, only mobility showed a significant difference in terms of disability level between the two groups (*p* < 0.05); moreover, although statistically nonsignificant, the relationship domain showed a higher disability level in the ABI group than that in the SCI group, with a p-value of 0.05, very close to statistical significance. In the group under 65 years old, the ABI group had a higher level of disability in the cognition and relationship domains, while the SCI group had a higher level of disability in the mobility domain (Table 3). 

As for onset duration (Table 4), for participants with onset duration shorter than 10 years, cognition revealed a higher level of disability in the ABI group, and mobility showed a higher level of disability in the SCI group. On the other hand, for participants with an onset duration of 10 years or more, the mobility domain showed a significantly higher level of disability in the SCI group (*p* < 0.05, Table 4). 

Linear-regression analysis revealed that onset duration was negatively correlated with cognition, relationships, participation, and summary scores of WHODAS 2.0 only for the ABI group (*p* < 0.05, Table 5), and there were no other significant associations.

Table 6 displays the OR for severe disability according to ICF classification using a multivariate-logistic-regression model. There were no significant relationships between severe disability and socioeconomic factors, including age, gender, area of residence, type of household, and marital, occupational, and economic status in either the ABI or SCI group (*p* > 0.05). 

## 4. Discussion

This study investigated differences in disability patterns between ABI and SCI in community-dwelling people with severe disability, and sought to identify risk factors related to disability. The SCI group experienced more difficulties with mobility, whereas the ABI group had more problems with cognition and relationships. Mobility was consistently more impaired in the SCI group than in the ABI group, irrespective of onset duration or age. Onset duration was negatively correlated with the cognition, relationships, participation, and summary scores of WHODAS 2.0 only in the ABI group. There were no significant relationships between socioeconomic factors and severity of disability in either group, which might mean that the type of disease is a crucial factor for the pattern of participation and activity in patients with severe disability. This study enhances our knowledge of the differences in function and disability between individuals with brain and physical impairments with severe disability. 

This study aimed to identify the pattern of functioning and disability in community-dwelling people with severe disability, and only enrolled participants with disability Grades 1 and 2 from the physical-disability and brain-impairment groups. Mean onset duration was more than 10 years for both groups, and the WHODAS 2.0 scores, excluding the domain of cognition, were below 50. 

Our results showed that the SCI group had higher disability in the mobility domain of WHODAS 2.0 than that of the ABI group, irrespective of age or onset duration. This is consistent with results of previous studies that compared TBI and SCI using the 12-item WHODAS 2.0 [16], and studies that reported mobility problems in SCI participants [26,27]. By contrast, a previous study found no difference in mobility between TBI and SCI groups [17]. The severity of disability in TBI participants compared to SCI patients may originate from the characteristics of enrolled participants. This study only included participants with severe disability with brain and physical impairments, and the SCI group showed a higher level of disability in mobility than the ABI. As expected, cognition and relationships showed a higher level of disability in the ABI group than that in the SCI group, which is consistent with previous studies [17,28].

There was a significantly different pattern of WHODAS 2.0 scores based on the participants’ age group. For participants under 65 years old, SCI patients revealed more difficulties with mobility and ABI patients had more problems with cognition and relationships. However, for participants 65 years old or older, the SCI group showed more disability in mobility compared to the ABI group, but there was no difference in cognition between the two groups. A previous animal study demonstrated that SCI induces chronic neuroinflammation and neurodegeneration in the brain, and is associated with cognitive decline [29]. In addition, a national cohort study in Taiwan suggested that participants with SCI had a significantly higher risk of dementia than participants without SCI [30]. SCI participants aged 65 years or older showed notably higher disability scores in cognition compared to the younger SCI participants (42.50 ± 22.52 vs. 30.59 ± 26.52, *p* < 0.05), which might demonstrate cognitive impairment after SCI. According to onset duration, mobility was only consistently impaired in SCI participants. 

In regression analysis for predicting WHODAS 2.0 scores, onset duration was significantly negatively related to cognition, relationships, participation, and summary scores in the ABI group; there were no other significant associations. More attention is warranted to preserve the functioning of ABI participants with regular follow-up and close observation. Although multivariate logistic regression sought to identify socioeconomic factors that might severely limit participation and activity, there were no significant risk factors associated with severe disability. Age, income level, and area of residence may be related to poor functioning in SCI patients [17,31], and age, gender, income level, and neighborhood socioeconomic status have been identified as significant predictors of functioning in stroke patients [19,32]. However, here, the type of disease itself rather than socioeconomic factors seemed to affect the patterns of participation and activity. 

This study has some limitations. First, our questionnaire did not collect information on comorbidities; however, comorbid diseases existing before the onset of a CNS injury and their later complications could influence functioning. Second, it was not possible to obtain information about the injury level and completeness of SCI; this survey was conducted in 65 PHCs involved in community-based rehabilitation programs and considered the type of disability, namely, brain or physical impairment, but detailed information on SCI was missing. Third, this was a cross-sectional study that cannot identify causal effects. Fourth, considering that our study participants visited the public health center, there is a possibility of selection bias, as our study sample may have had a higher level of activity and participation than the general ABI and SCI population. We did not specify the relevant parameters, such as cluster type or homogeneity, which could affect the dependent variable. Fifth, missing and invalid data could also cause selection bias; participants who had more difficulties in completing the whole questionnaire may also have more severe problems in communication and cognitive concentration. Sixth, the effectiveness of this population-based survey study might be limited to Korea. Differences in disability grading systems, and cultures between countries may cause differences in injury-related functional disability status. Lastly, differences of participation and activation according to gender were not investigated in this study. Future longitudinal follow-up studies with more detailed medical conditions are warranted.

However, despite these limitations, one strength of our research is that it presents the results of a survey reporting the activity and participation of people with severe CNS injuries at the national level in Korea for the first time using WHODAS 2.0.

## 5. Conclusions

This study identified the different patterns of disability according to CNS injury type in patients with severe disability. SCI patients consistently exhibited a higher level of disability in mobility than ABI patients. Onset duration negatively affected overall participation and activity level in the ABI group. The type of disease seemed to be a crucial factor for the pattern of participation and activity in patients with severe disability. Understanding the differences in the pattern of disability between these two groups of community-dwelling people with severe disability may help to establish appropriate management of health resources. Healthcare plans regarding transportation and accessibility problems for SCI patients need to be re-evaluated. Implementation of cognitive-rehabilitation programs with close follow-up and regular evaluations for elderly individuals with CNS injuries are warranted. 

## Figures and Tables

**Table 1:** General characteristics of participants (*N* = 505).

Characteristics	Categories	Acquired Brain Injury (*N* = 322)	Spinal-Cord Injury (*N* = 183)	*p*-Value
Sex, *N* (%)	Male	188 (58.6%)	107 (59.1%)	0.904
	Female	133 (41.4%)	74 (40.9%)	
Age, *N* (%)	<65	188 (59.5%)	127 (70.2%)	0.017
	≥65	128 (40.5%)	54(29.8%)	
Duration of disability, year (SD)		10.71 (7.72)	19.36 (14.70)	0.001
Area of residence, *N* (%)	Metropolitan city	45 (14.0%)	8 (4.4%)	0.001
	Non-metropolitan city	53 (16.5%)	54 (29.5%)	
	Rural	223 (69.5%)	121 (66.1%)	
Type of household	Apartment	112 (34.8%)	79 (43.2%)	0.062
	Others	210 (65.2%)	104 (56.8%)	
Marital status, *N* (%)	Never married	29 (9.1%)	44 (24.0%)	0.001
	Currently married	210 (66.0%)	98 (53.6%)	
	Separated	79 (24.8%)	41 (22.4%)	
Occupational status, *N* (%)	Employed	9 (2.8%)	9 (5.0%)	0.010
	Unemployed	279 (87.7%)	162 (90.5%)	
	Retired	26 (8.2%)	3 (1.7%)	
	Student	4 (1.3%)	5 (2.8%)	
Economic state, *N* (%)	High	4 (1.3%)	2 (1.1%)	0.003
	Middle	127 (40.7%)	46 (25.7%)	
	Low	181 (58.0%)	131 (73.2%)	

**Table 2:** Comparison of World Health Organization Disability Assessment Schedule 2.0 (WHODAS 2.0) scores of overall disability across domains between participants with brain and spinal-cord injuries.

	Acquired Brain Injury (*N* = 322)	Spinal-Cord Injury (*N* = 183)	*p*-Value
Cognition	44.27 ± 28.47	34.29 ± 25.75	<0.0001
Mobility	62.81 ± 29.81	75.76 ±28.23	<0.0001
Self-care	54.60 ± 29.00	55.41 ± 32.05	0.771
Relationships	53.83 ± 30.97	45.08 ± 31.00	0.002
Life activities	71.58 ± 30.59	73.77 ± 28.89	0.432
Participation	61.00 ± 26.76	61.20 ± 24.92	0.927
Summary score	57.20 ± 23.54	56.52 ± 21.16	0.733

**Table 3:** Comparison of WHODAS 2.0 scores of overall disability across domains between participants with brain and spinal-cord injuries according to age.

Mean (SD)	<65 years			≥65 years		
	Acquired Brain Injury (*N* = 188)	Spinal-Cord Injury (*N* = 127)	*p*-Value	Acquired Brain Injury (*N* = 128)	Spinal-Cord Injury (*N* = 54)	*p*-Value
Cognition	43.35 ± 29.60	30.59 ± 26.52	<0.001	45.16 ± 26.73	42.50 ± 22.52	0.523
Mobility	60.49 ± 30.95	75.76 ± 29.20	<0.001	65.58 ± 27.94	75.09 ± 26.72	0.035
Self-care	54.15 ± 29.95	56.54 ± 33.10	0.507	55.08 ± 27.75	52.22 ± 29.76	0.536
Relationships	52.93 ± 32.14	44.73 ± 31.86	0.027	55.15 ± 29.26	45.70 ± 30.17	0.050
Life activities	71.38 ± 30.72	74.57 ± 29.03	0.357	71.41 ± 30.24	71.48 ± 29.29	0.980
Participation	61.85 ± 26.98	62.02 ± 26.41	0.957	59.63 ± 26.59	59.78 ± 20.94	0.970
Summary score	56.66 ± 24.52	56.08 ± 22.15	0.830	57.71 ± 26.59	57.26 ± 19.33	0.897

**Table 4:** Comparison of WHODAS 2.0 scores of overall disability across domains between participants with brain and spinal-cord injuries according to onset duration.

Mean (SD)	<10 years			≥10 years		
	Acquired Brain Injury (*N* = 172)	Spinal-Cord Injury (*N* = 41)	*p*-Value	Acquired Brain Injury (*N* = 142)	Spinal-Cord Injury (*N* = 130)	*p*-Value
Cognition	50.76 ± 29.57	39.27 ± 26.14	0.023	37.29 ± 25.76	32.04 ± 25.70	0.094
Mobility	64.43 ± 29.36	78.76 ± 28.17	0.005	60.97 ± 30.74	74.78 ± 29.01	<0.001
Self-care	59.53 ± 28.86	63.17 ± 32.82	0.481	49.30 ± 28.45	53.23 ± 32.28	0.286
Relationships	60.16 ± 30.11	50.24 ± 33.15	0.065	45.87 ± 30.60	43.45 ± 30.79	0.515
Life activities	75.35 ± 27.86	79.76 ± 27.34	0.362	67.32 ± 33.54	72.77 ± 29.33	0.157
Participation	65.47 ± 25.12	67.83 ± 25.25	0.589	55.61 ± 28.38	60.10 ± 25.03	0.169
Summary score	61.84 ± 22.86	62.07 ± 21.58	0.952	51.89 ± 23.93	54.97 ± 21.54	0.268

**Table 5:** Linear regression analyses with WHODAS 2.0 scores as the dependent variable.

Coefficients	Acquired Brain Injury (*N* = 322)		Spinal-Cord Injury (*N* = 183)	
	Age	Onset Duration	Age	Onset Duration
Cognition	0.059	−0.234 *	0.121	0.049
Mobility	0.084	0.014	0.115	0.040
Self-care	0.027	−0.108	−0.017	−0.056
Relationships	0.039	−0.170 *	−0.077	0.006
Life activities	0.007	−0.031	−0.001	0.025
Participation	−0.007	−0.168 *	−0.030	−0.082
Summary score	0.042	−0.155 *	0.031	−0.010

* *p* < 0.05.

**Table 6:** Factors associated with severe disability measured using International Classification of Functioning, Disability, and Health (ICF) in multivariate logistic regression.

Variable	Acquired Brain Injury			Spinal-Cord Injury		
	OR	95% CI	*p*-Value	OR	95% CI	*p*-Value
Age (<65years)						
≥65	1.394	0.816–2.384	0.4717	1.191	0.560–2.534	0.6496
Sex (male)						
Female	0.723	0.433–1.205	0.2244	0.852	0.427–1.699	0.6495
Area of residence (Metropolitan city)						
Nonmetropolitan city	2.398	0.967–5.947	0.0568	2.409	0.513–11.307	0.2886
Rural	1.288	0.643–2.583	0.5018	2.028	0.452–9.096	0.5677
Type of household (Apartment)						
Others	1.128	0.668–1.905	0.6529	0.965	0.462–2.015	0.9240
Marital Status (Never married)						
Currently married	0.872	0.322–2.362	0.8764	0.833	0.331–2.096	0.8227
Separated	0.688	0.242–1.952	0.3877	0.815	0.286–2.326	0.7898
Occupational Status (Retired)						
Employed	0.377	0.067–2.106	0.5897	0.115	0.014–3.061	0.0592
Unemployed	0.657	0.246–1.757	0.5486	1.408	0.113–17.557	0.2707
Student	0.314	0.029–3.421	0.5446	2.345	0.076–72.096	0.2688
Economic state (Low)						
High	0.140	0.014–1.435	0.1427	–		
Middle	0.623	0.371–1.045	0.4127	0.709	0.327–1.534	0.9542

## Supplementary Materials

The following are available online at https://www.mdpi.com/1660-4601/17/9/3031/s1. Table S1: ICD-10 codes for acquired brain and spinal-cord injuries.
