# Supplementary material for: Comparing Activity and Participation between Acquired Brain Injury and Spinal-Cord Injury in Community-Dwelling People with Severe Disability Using WHODAS 2.0
Source: Int J Environ Res Public Health. 2020 Apr 27;17(9):3031. doi: 10.3390/ijerph17093031 (PMC7246687; doi:10.3390/ijerph17093031)
Supplement: Supplementary file 1 [file ijerph-17-03031-s001.pdf]

ICD-10 codes for acquired brain injuries and spinal cord injuries	
	Acquired brain injuries
I60-I62	Hemorrhagic stroke
I63	Cerebral infarction
I64	Stroke, not specified as haemorrhage or infarction
S06	Intracranial injury (without S06.0)
G20	Parkinson's disease
G931	Anoxic brain damage, not elsewhere classified
	Spinal cord injuries
G95.9	Disease of spinal cord, unspecified
M47.1	Other spondylosis with myelopathy
S14	Injury of nerves and spinal cord at neck level
S14.0	Concussion and edema of cervical spinal cord
S14.1	Other and unspecified injuries of cervical spinal cord
S24	Injury of nerves and spinal cord at thorax level
S24.0	Concussion and edema of thoracic spinal cord
S24.1	Other and unspecified injuries of thoracic spinal cord
S34	Injury of lumbar and sacral spinal cord and nerves at abdomen, lower back and pelvis level
S34.0	Concussion and edema of lumbar and sacral spinal cord
S34.1	Other and unspecified injury of lumbar and sacral spinal cord
